# The greenhouse as an integrated management strategy for *Prodiplosis longifila* Gagné, 1986: effects on infestation and pesticide use

**DOI:** 10.1186/s12870-026-08368-2

**Published:** 2026-02-25

**Authors:** Maria Esmeralda Cuzco, Amalia Marisol Vera, Maria Leticia Vivas, John Eloy Franco, Maria Teresa Lao

**Affiliations:** 1 Carrera de Agronomía, Facultad de Ciencias Agrarias, Universidad de Guayaquil, Av. Las aguas y Juan Tanca Marengo, Guayaquil, 090112 Ecuador; 2 Carrera de Agropecuaria, Facultad de Educación Técnica para el Desarrollo, Universidad Católica de Santiago de Guayaquil, Av. C. J. Arosemena Km. 1.5, Guayaquil, 09014671 Ecuador; 3 Departamento de Agronomía, Escuela Superior de Ingeniería, CIAIMBITAL, Campus de Excelencia Internacional Agroalimentario (ceiA3), Universidad de Almería, Ctra. Sacramento s/n, Almería, 04120 España

**Keywords:** Integrated pest management, population dynamics, Sampling methods, Monitoring, Pesticide efficiency

## Abstract

**Background:**

Tomato production on the Ecuadorian coast is primarily carried out in open fields and has declined due to infestation by *Prodiplosis longifila*. Its short life cycle and rapid recolonization necessitate intensive chemical control, generating selection pressure and environmental risks. *P. longifila* exhibits high ecological plasticity and, due to its potential for expansion, has been included by the European and Mediterranean Plant Protection Organization (EPPO) and the European Food Safety Authority (EFSA) on the list of priority pests, with a risk of establishment in temperate regions of Europe and coastal areas of the Mediterranean.

Given the urgent need for sustainable strategies to revitalize tomato production, the objective of this study was to evaluate the impact of greenhouse use by comparing infestation levels, population density, the effectiveness of sticky traps, and pesticide efficacy in two cultivation systems: open fields and greenhouses.

**Results:**

The population dynamics of *Prodiplosis longifila *varied significantly among the cultivation systems. In greenhouse, infestation above the threshold ranged from 13% to 21.9%, while in open field it reached critical levels of 61.3%. Monitoring adults using traps is not a good indicator of abundance. Pesticide efficacy varies depending on the environment and infestation level. In greenhouse, the biorational insecticides spinetoram and abamectin achieved efficacies exceeding 80% in infestations of 10% to 20%, *Azadirachta indica* caused burns at high temperatures, and the efficacy of abamectin decreased. In open field, the neonicotinoids acetamiprid and imidacloprid achieved efficacies of up to 90% in extreme infestations (50%–61%), and the efficacy of thiamethoxam + lambda-cyhalothrin decreased after the third application.

A positive correlation was observed between temperature and larval density, while relative humidity had a negative effect. The results demonstrate that direct monitoring is an effective tool for optimizing the number of applications and that anti-aphids mesh is a physical barrier to reduce infestation, allowing for the rational use of pesticides.

**Conclusion:**

The use of anti-aphid mesh in greenhouse significantly reduces infestation by *P. longifila*, decreases the frequency of spraying, and promotes the selection of biorational pesticides. This positions greenhouses as an alternative for revitalizing tomato production in areas affected by this pest. The results are relevant for tropical contexts and provide valuable information for designing prevention and management policies in at-risk regions.

**Graphical Abstract:**

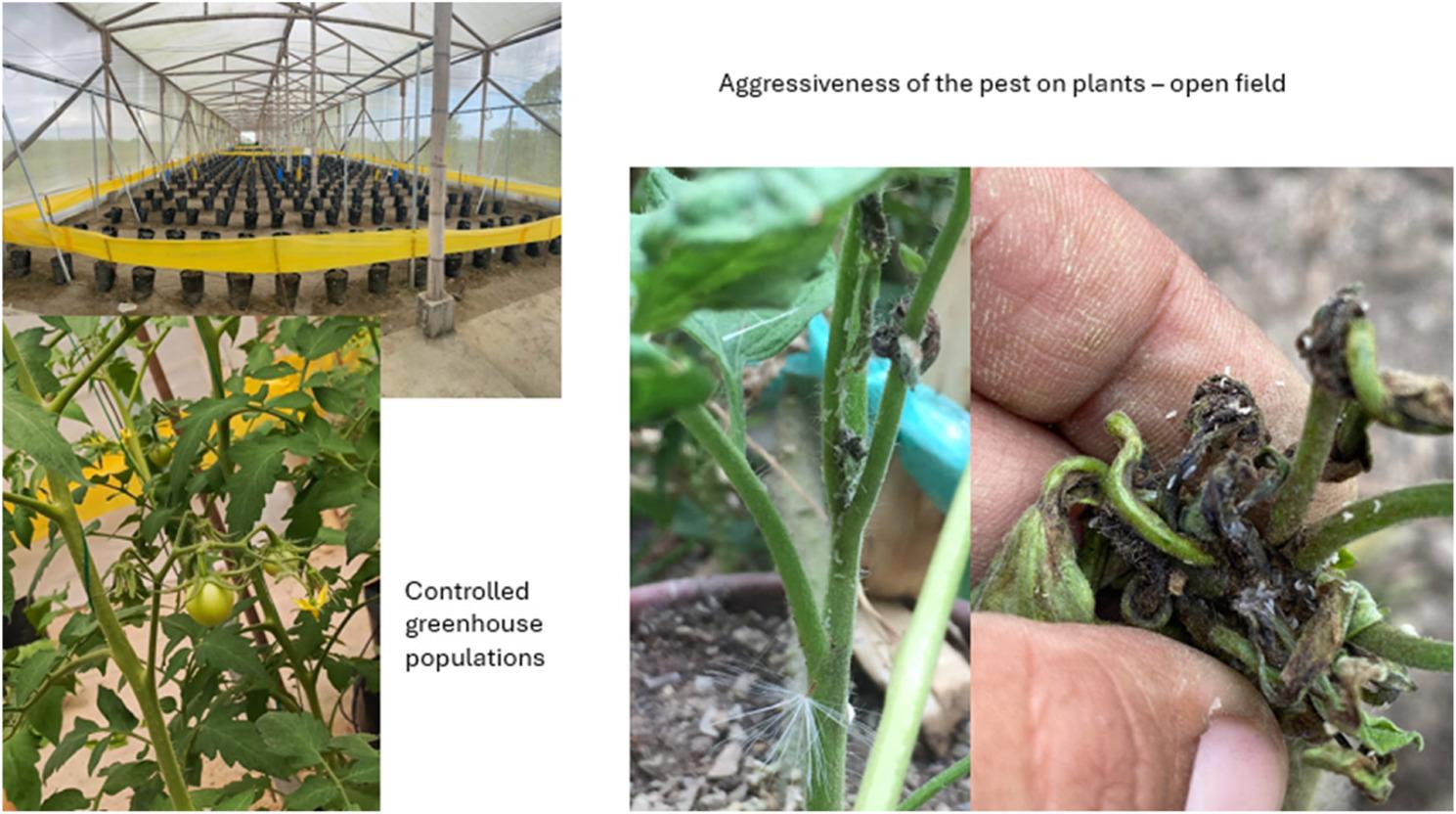

## Background

*Prodiplosis longifila* Gagné, 1986, belonging to the family Cecidomyiidae and commonly known as “negrita,” is a polyphagous pest that causes severe damage to tender shoots and inflorescences, potentially leading to premature abscission of flowers and small fruits [1]. The genus *Prodiplosis* is established in both the Americas and Europe. Nine species have been reported in South America, while others are known in Europe, causing significant losses in crops such as potato (*Solanum tuberosum* L.), tomato (*Solanum lycopersicum* L.), and melon (*Cucumis melo* L.) [2]. In Peru, it causes considerable losses in asparagus (*Aparagus officinalis* L.) [3] and potato crops, where infestations have reached up to 16% of the buds. In Florida, USA, it affected up to 25% of the flower buds of Tahiti lime (*Citrus x latifolia* Tanaka ex Q. Jimenez) [4]. In Bolivia and South Africa, it has been utilized for weed control, particularly against *Jatropha* sp. and other species of the Euphorbiaceae family [5].Peña et al. [1] report its presence in the West Indies and Jamaica. *P. longifila* has also been documented in chili pepper (*Capsicum frutescens* L.), bell pepper (*Capsicum annuum* L.), and other companion plants [6]. In Ecuador, Valarezo et al. [7] confirmed its presence in 12 provinces across both the coastal and highland regions up to 1,800 m above sea level, affecting crops in both open field and greenhouse conditions. *P. longifila* has a wide range of hosts, including tomato, potato, beans, melon, cucumber, cotton, castor bean, and pepper. Nevertheless, with tomato being the host where it has shown the greatest aggressiveness, potentially causing losses of up to 100% of the production in the coastal region of Ecuador. In Guayas province, in 2021, 170 hectares of tomato were cultivated, yielding 3,857 t, which corresponds to a productivity of 2.3 kg/m^2^ [8]. In 2022, the same area yielded only 1.2 kg/m^2^ [9].Valarezo et al. [7] mentions that this situation has led to the indiscriminate use of pesticides, characterized by frequent and inadequate applications, following fixed spraying schedules. Furthermore, they estimate that between 45% and 80% of producers resort to agrochemicals, making between 21 and 30 applications per crop cycle. Organophosphates are the primary group used to control this pest [7], while some farmers have abandoned the crop altogether. Therefore, one of the strategies employed in Integrated Pest Management (IPM) is the use of biorational insecticides, which play a critical role in medium- and long-term agricultural sustainability [10].

The ecological niche model of *P. longifila* is constrained by altitude, temperature, and precipitation [11]. These authors highlighted that warm regions favor the presence and development of *Prodiplosis longifila*, although the species shows a remarkable capacity to adapt to temperate zones. However, climatic variables such as precipitation and high relative humidity tend to negatively influence its infestation level. Furthermore, Duque et al. [12] reported an increase in the number of larvae after insecticide applications, suggesting a possible resurgence effect associated with the reduction of natural enemies or sublethal doses. However, Cañarte et al. [13] suggest that the dry season is most favorable for *P. longifila* populations, recommending planting at the end of the rainy season (April) when pest populations are low [7]. In addition, viviparity in *P. longifila* has been observed in the coastal region of Ecuador, where females deposit first-instar larvae directly [7]. This was confirmed by dissecting female reproductive organs and finding larvae inside. Finally, these authors mention that the insect’s life cycle includes a first-stage larva lasting 2.55 days, a second-instar larva lasting 2.70 days, a third-instar larva lasting 2.87 days, a prepupal stage of 1.50 days, a pupal stage of 6.35 days, and an adult longevity of 1.35 days. In Colombia, however, females lay eggs on leaf and flower buds beneath the calyx, and once hatched, larvae begin feeding on the epidermal tissues of the buds [11]. This behavior is due to the adaptive evolution of insects because in viviparous species there is an enrichment of genes due to greater selection pressure from resource allocation [14]. Since tomato is the primary crop affected by *P. longifila*, the European and Mediterranean Plant Protection Organization (EPPO) has included this pest on its alert list due to the risks it poses to the region [15]. This organization highlights the potential of this pest to establish in the Mediterranean Basin, Portugal, and the southern Black Sea coasts. It could also establish, albeit with lower probability and greater uncertainty, in oceanic climate regions of Western Europe, such as western France and southern United Kingdom [16].

Geraud et al. [17] attribute pesticide dependency to limited or nonexistent bioecological knowledge, which hinders the rational management of *P. longifila* and exacerbates the problem. In many cases, this abandonment led to a drastic reduction in tomato production in the coastal region, prompting many farmers to switch to other crops. In response to this issue, greenhouse systems emerge as a viable alternative to revitalize production, providing more controlled conditions that could facilitate more effective pest management. However, there is limited information on the comparative efficacy of phytosanitary treatments in greenhouses and open fields, as well as on the influence of climatic factors on the population dynamics of *P. longifila* and the effectiveness of pesticides.

In this context, the present study aims to evaluate the impact of greenhouse management as a strategy to reduce *P. longifila* infestation and restore tomato production in the region. To achieve this, infestation levels in plants treated with different pesticides were compared between greenhouse and field condition, the infestation in untreated plants was analyzed, and the relationship between climatic parameters, larval density, and their effect on pesticide efficacy was examined. The findings of this study will provide key information to optimize the management of this pest and promote more effective strategies for restoring tomato cultivation in the Coastal region. In this sense, the study generates information based on the real management conditions and the natural pressure of the pest, which is relevant for the analysis of integrated management of *P. longifila* in tomato production systems of the region.

## Materials and methods

### Study area

This research was conducted in San Isidro, Guayas Province, Ecuador. The Coastal region is characterized by a Tropical Megathermal climate, classified as humid, with excessive rainfall during the rainy months, necessitating the use of protective structures for short-cycle crops. Conversely, dry periods require the implementation of irrigation systems to ensure optimal crop development. The average temperature ranges between 20 °C and 31 °C, with maximum values occurring between February and April and minimum values between August and September [18]. The mean relative humidity reaches its highest values in February and March at 84.8%, while the lowest values are recorded in August and September at 81.9%. The soil is clay-loom with low water permeability and high water and nutrient retention capacity. It has an electrical conductivity of 0.40 dS m⁻¹, a cation exchange capacity of 37.0 (moderate to high), a pH of 6.7, and an organic matter content of 2%.

The study was carried out during the dry season from May to October, as these months exhibit the highest infestation levels of various pests.

### Experimental design

The trials were conducted both in greenhouse and open field conditions, using the tomato variety Zodiac. This variety is indeterminate and resistant to tomato yellow leaf curl virus (TYLCV), nematodes, *Fulvia fulvia*, and tomato mosaic virus [19].

#### Greenhouse setup

The experiment was performed in a 300 m² section of a 1,000 m² greenhouse adapted to tropical regions. The greenhouse structure was made of native bamboo (*Guadua angustifolia* Kunth) with a gutter height of 4.10 m, a ridge height of 6.35 m, and 5 m between pillars. It included a ridge vent for ventilation. The perimeter surface and ridge vent were covered with a mesh of 26 × 40 filaments inch (caliber 12), covering 47% of the greenhouse surface. The cover film was a 204 μm transparent plastic with three layers: external layer (30% Metallocene Low Linear Density Polyethylene (mLLDPE), 62.5% Low Density Polyethylene (LDPE), 7.5% Hindered Amine Light Stabilizers (HALS)-type UV additive), intermediate layer (30% mLLDPE, 62.5% LDPE, 7.5% HALS-type UV additive), and inner layer (100% LDPE).

The experiment followed a Randomized Complete Block Design (RCBD) in two environments (open field and greenhouse). Each environment was treated as an independent experimental condition, with 306 plants in each environment. The planting density was 0.50 m between plants and 1.20 m between rows. In the greenhouse, the plants were cultivated in 1 L bags containing a substrate composed of river sand, rice husk, and topsoil (50:25:25 v/v). Irrigation emitters with a flow rate of 4.7 L h⁻¹ were used for a dosage of 7 min. The nutrient solution, applied via a Venturi system throughout the crop cycle consisted of 15 mM NO₃⁻, 2 mM SO₄²⁻, 1 mM H₂PO₄⁻, 6 mM K⁺, 4 mM Ca²⁺, 2 mM Mg²⁺, with a pH of 6 and an electrical conductivity (EC) of 2 dS m⁻¹ [20].

#### Open field setup

In the open field, land preparation followed the same planting density, irrigation type, and fertilization protocol. In both environments, cultural practices such as pruning, staking, weeding and border maintenance were implemented to reduce pest establishment.

The tomato crops in both environments were left to natural pest infestation. Monitoring was conducted twice per week to determine infestation thresholds and facilitate decision-making. Pest control was implemented either broadly or in specific areas, depending on pest progression and the strategy adopted, following Integrated Pest Management (IPM) principles, where chemical control was used as a last resort.

### Determined parameters

#### Climatic parameters

Temperature (°C) and relative humidity (%) were recorded every 15 min using an Elitech 6G Data Logger Thermohygrometer (USA), calibrated with buffered probe solutions for glycol bottles and positioned in the center of the crop. To correlate the infestation percentages of treated and untreated plants and the number of larvae under both greenhouse and open field conditions, mean temperature, maximum temperature, and relative humidity were calculated.

#### Infestation percentage

To determine treatment thresholds in both environments (greenhouse and open field), direct observations were conducted at fixed sampling points, evaluating 25 plants per repetition twice a week. Five random shoots or flowers were selected from each plant, excluding edge plants to minimize data bias. A binomial sampling criterion was applied, where the presence or absence of live larvae was recorded on each plant, as the presence of larvae, regardless of their number, indicates infestation. This binomial approach is commonly used in pest monitoring studies for a quick and efficient assessment of infestation levels and decision-making.

The equation proposed by Valarezo et al. [7] was used to calculate infestation levels.

$$\:\%I=\left(\frac{BI}{BM}\right)*100$$

Where:

%I: Infestation percentage.

BI: Infested shoots.

BM: Sampled shoots.

For untreated plants, fixed sampling points were used, where the number of larvae on each plant was counted to provide a more precise quantitative estimate of infestation intensity in the absence of control. Sampling was carried out by observing 10 plants per repetition, totaling 30 plants (10 per repetition) per environment (greenhouse and open field) in each evaluation. These evaluations were also conducted twice a week, randomly selecting ten shoots or flowers on each plant. Infestation assessments and larval counts were performed simultaneously on each sampling date, with the same frequency throughout the experimental period.

#### Population Estimation through monitoring with chromatic traps

**Fig. 1:**
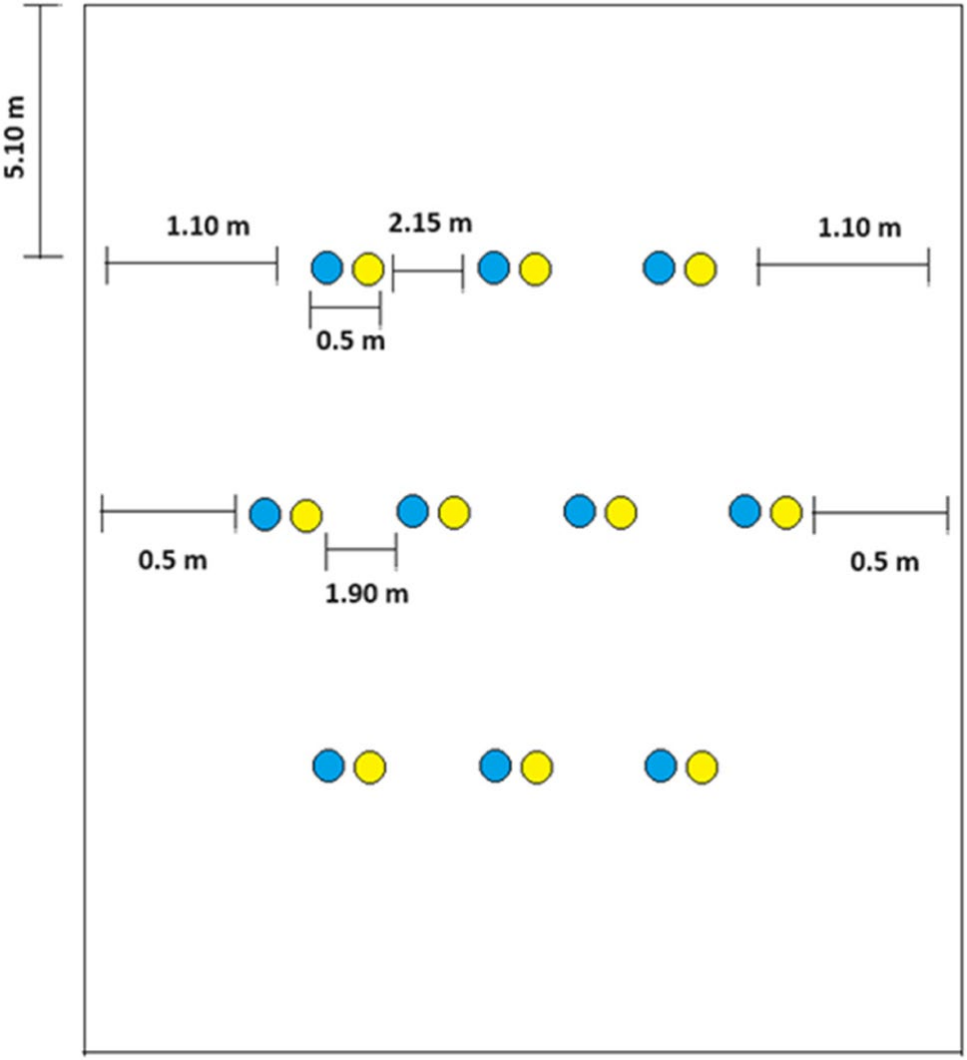
Distribution of yellow and blue traps in open field and greenhouse conditions

The monitoring of *P. longifila* adults was conducted using yellow and blue chromatic traps measuring 30 cm × 10 cm, deployed at the beginning of the crop cycle. These traps were placed 20 cm above the plant canopy in a staggered arrangement, as shown in Fig. 1. The traps were replaced weekly, and the number of adults captured was recorded. The trap height was adjusted as the crop grew to maintain optimal coverage.

Yellow traps were selected due to their high attraction to dipterans and other phytophagous insects, while blue traps were included to assess their effectiveness in capturing cecidomyiids, as some studies have reported significant dipteran captures using this color [21–24].

#### Applied treatments

Following the principles of Integrated Pest Management (IPM), treatments were applied whenever the treatment threshold of 10% infestation was exceeded [7]. Various treatments were conducted in both greenhouse and open field conditions throughout the crop cycle, starting with products of lower toxicological categories (Table 1).

**Table 1:**
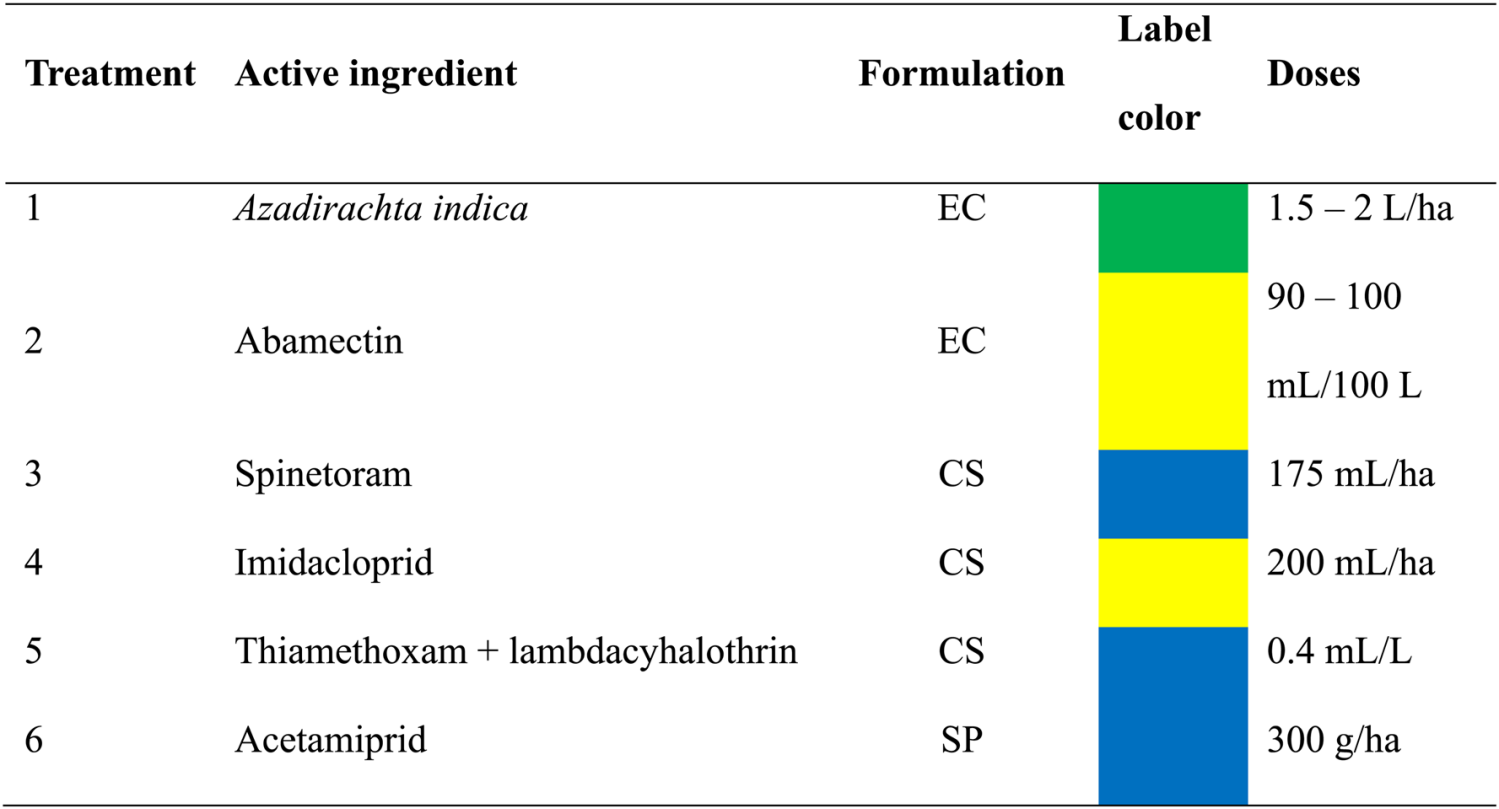
Products used in the trials, listed from lowest to highest toxicological category and label color

Emulsifiable concentrate (EC), Concentrated suspension (CS), Soluble powder (SP). Colors are assigned according to the World Health Organization (WHO) toxicological classification

The larvae of *P. longifila* are characterized by scraping and piercing the young tissues of tomato plants. Therefore, the pesticides used in this study were selected based on their reported efficacy against *P. longifila*, their compatibility with Integrated Pest Management (IPM), and the need to implement a rotation program to prevent the development of resistance. Botanical products, bioinsecticides, and synthetic compounds with different modes of action were included in order to assess their impact on the pest under greenhouse and open field conditions.

*Azadirachta indica* is a botanical insecticide with translaminar action that disrupts development and inhibits feeding [25]. Abamectin is a semi-synthetic translaminar insecticide with neurotoxic action that causes paralysis and death in insects [26]. Spinetoram is a semi-synthetic insecticide obtained from spinosins with translaminar action that acts on nicotinic receptors and gamma-aminobutyric acid (GABA) [27, 28]. Imidacloprid is a systemic neonicotinoid that interferes with nicotinic acetylcholine receptors in the central nervous system of insects [29]. Thiamethoxam + lambdacyhalothrin is a systemic neonicotinoid and broad-spectrum pyrethroid combination that provides a knockdown effect [30, 31]. Acetamiprid is a systemic neonicotinoid with translaminar activity, characterized by rapid absorption in the plant [31, 32].

A manual backpack sprayer equipped with a full-cone nozzle was used for pesticide application. The application frequency was determined based on treatment thresholds, with treatments conducted only when infestation levels reached the established threshold. The pesticide doses were adjusted according to the manufacturer’s recommendations and spraying took place between 4:00 and 5:30 p.m.

#### Efficacy of treatments

Abbott’s efficacy formula will be used to determine the effectiveness of the treatments [33]. The effectiveness of the different pesticides was calculated using the following formula:

$$\mathrm{Efficacy}\left(\%\right)=\frac{\left(\text{Initial Population - Post-treatment Population}\right)\times 100}{\text{Initial Population}}$$

The initial population is sampled before the treatment is applied, and the post-treatment population is sampled during the following evaluation.

#### Determination of pest population dynamics

To estimate the pest population dynamics, five plants were randomly selected from each repetition twice a week. On each plant, 10 shoots and/or flowers were evaluated, and the number of larvae from all larval stages present on the different substrates was counted.

### Statistical analysis

The data are presented as means, percentages, standard deviations, standard errors, and estimates. Statistical analyses were performed using RStudio version 4.2.3, employing various packages such as dplyr, tidyr, ggplot2, psych, ggpubr, emmeans, lmerTest, and agricolae. A Student’s t-test for independent samples was conducted to estimate significant differences between the various variables, as well as between greenhouse and field environments.

To explore the relationship between climatic parameters and infestation in treated and untreated plants, a Generalized Additive Model (GAM) was used, as it allows for capturing nonlinear relationships and complex patterns in the data, such as peaks or asymmetric behaviors, thus enabling visualization of the relationship between predictors (climatic parameters) and the response variable (infestation).

To explore the relationship between climatic parameters and infestation in treated and untreated plants, a combination of Generalized Additive Models (GAM) and Generalized Mixed Linear Models (GLMM) was used. GAM was employed for exploratory purposes, allowing the identification of nonlinear relationships and complex patterns between climatic predictors and infestation, as well as the visualization of asymmetric responses, thresholds, and peaks that are not always adequately captured by strictly parametric models. Together, this approach allowed GAM to characterize the shape of the relationship between variables, while GLMM provided robust quantitative estimates of how infestation probability and larval density increase or decrease between environments and along climatic gradients, expressed in ecologically interpretable terms.

For statistical inference, global infestation measurement models (GLMM) were fitted to quantify the magnitude and direction of the effects of the cropping system and climatic variables on infestation. In treated plants and in the analysis of larval numbers, data were modeled using a GLMM with a Tweedie distribution, considering the percentage of infestation and the number of larvae as response variables, respectively. The model included the cropping system, climatic parameters, and their interactions as fixed effects, allowing for the evaluation of differences in the thermal response of infestation between environments. These evaluations were incorporated as a random effect to control for temporal dependence between repeated observations.

In untreated plants, a Hurdle-Binomial model, suitable for data with a high proportion of structural zeros, was used. This model explicitly discriminates between two distinct biological processes: (i) the probability of infestation and (ii) the response once an infestation is established. The response variable was the presence of infestation, while the cropping system, climatic parameters, and their interactions were included as fixed effects. This allowed for the evaluation of differences in both the baseline level of infestation and sensitivity to thermal extremes between systems. Evaluations were incorporated as a random effect to control for dependence between repeated observations over time.

All GLMMs were adjusted using logarithmic links, so the coefficients were initially estimated on the link scale. To facilitate ecological interpretation, the coefficients associated with continuous climatic variables were transformed to the original response scale using the exponential function. These effects were interpreted as multiplicative changes, expressed as relative percentage variations in infestation intensity or infestation probability for each unit increase in the explanatory variable. Since infestation was assessed in two contrasting production environments (open field and greenhouse), with clearly differentiated microclimates, interactions between the environment and climatic variables were included. This allowed for the estimation of slopes specific to each cropping system and acknowledged that the effect of climate can vary in both magnitude and direction between environments.

To evaluate the efficacy of pesticides applied to tomatoes under greenhouse and field conditions, a generalized linear mixed model (GLMM) with a Tweedie distribution was fitted using the `glmmTMB` function. The response variable was the efficacy of each pesticide, expressed as a percentage reduction in infestation. Fixed effects included the type of pesticide used and the infestation level of the treated plants at the time of application, to control the effect of initial pest density on efficacy. The evaluation was included as a random effect to account for the hierarchical structure of the data.

Residue diagnostic tests were performed to verify the model assumptions using the DHARMa package. Homoscedasticity and dispersion were assessed using the nonparametric dispersion test (DHARMa). Residue uniformity was verified using the Kolmogorov-Smirnov test (testUniformity). In addition, zero inflation was assessed using the DHARMa zero inflation test, verifying that the observed proportion of zeros matched the expected proportion according to the fit.

## Results

### Climatic parameters

In Table 2 are presented various parameters that define the climatic conditions of the trial environment.

**Table 2:** Average and coefficient of variation (CV) of Climatic parameters: absolute minimum and maximum Temperature; minimum, mean and maximum temperature; mean relative humidity and absolute relative humidity

Parameters	Greenhouse		Field		
	Average	CV (%)	Average	CV (%)	
A Min T (°C)	20.6	3.97	18.5	8.11	
Min T (°C)	21.6	3.78	20.2	7.43	
Mean T (°C)	26.3	3.95	25.2	3.84	
Max T (°C)	34.4	6.87	32.1	5.04	
A Max T (°C)	41.1	5.75	37.4	4.33	
Mean RH (%)	79.4	3.12	81.6	4.75	
A RH (%)	83.0	2.98	86.3	4.49	

**Fig. 2:**
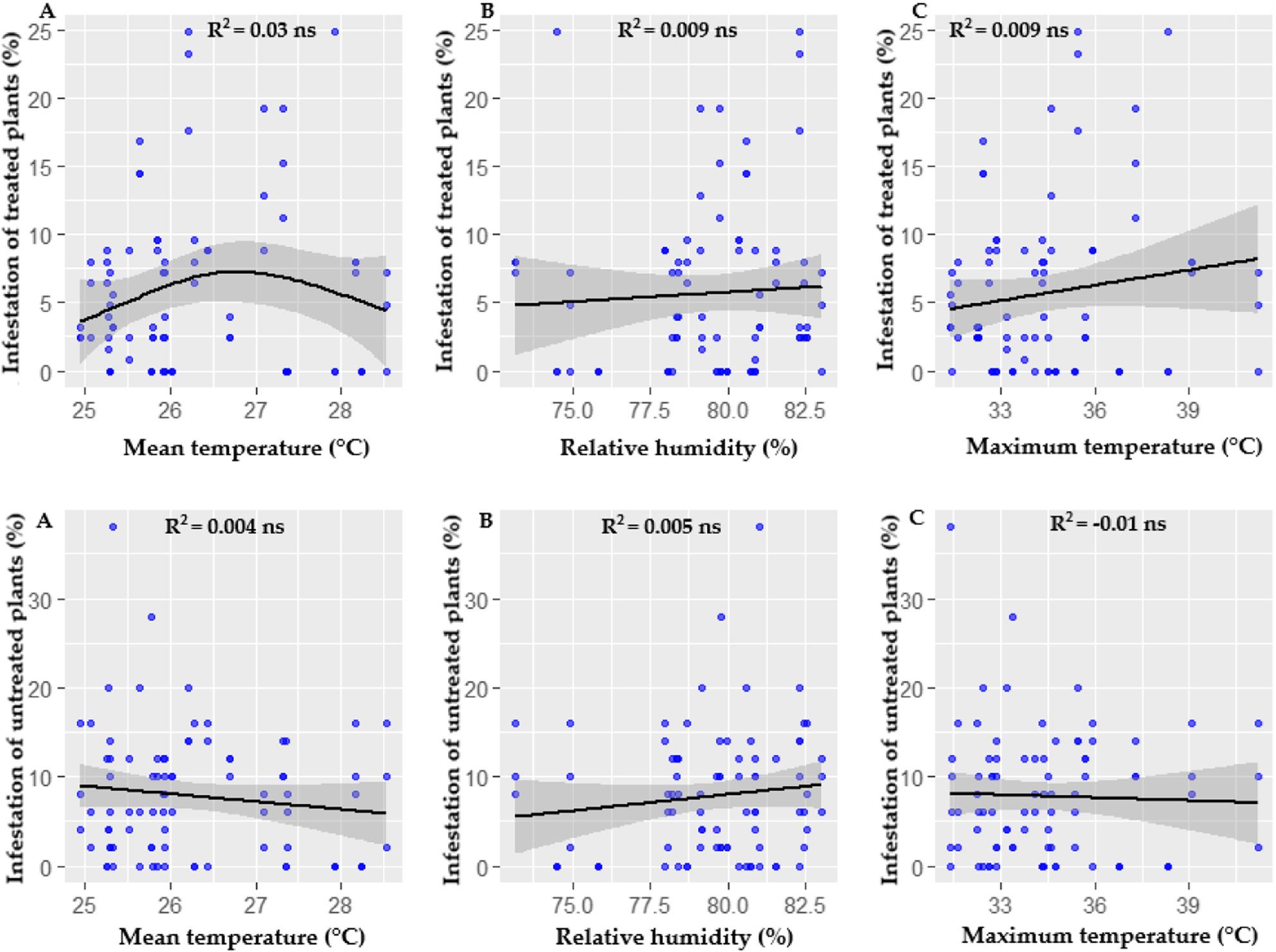
Relationship between climatic factors and the infestation percentage in greenhouse. Infestation of treated and untreated plants: **A** Mean temperature. **B** Relative humidity. **C** Maximum temperature

Under greenhouse conditions, infestation by *P. longifila* did not show a significant relationship with variations in the climatic parameters evaluated, in both treated and untreated plants (Fig. 2).

**Fig. 3:**
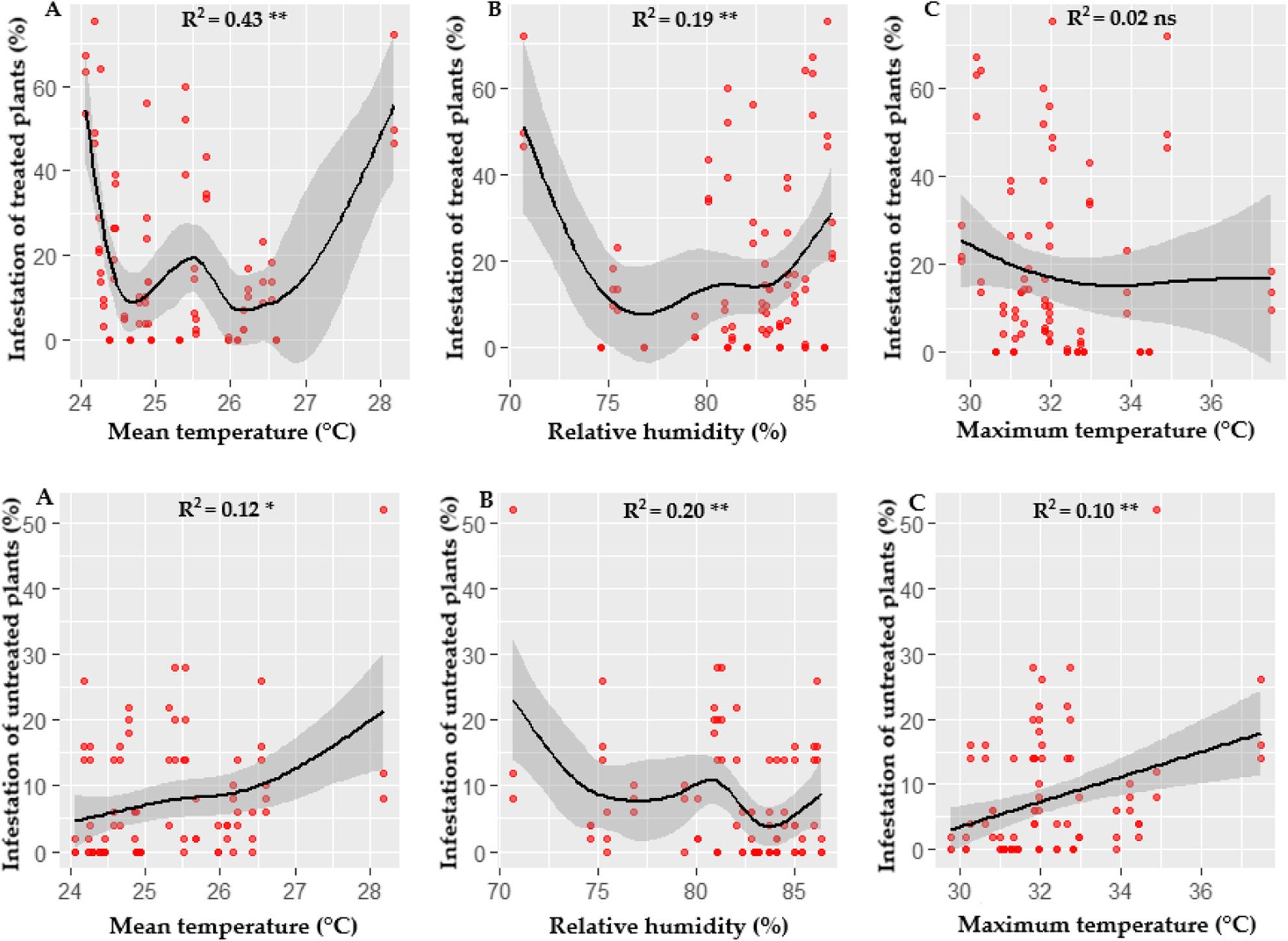
Correlation between climatic factors and infestation percentage in open field conditions. Infestation of treated and untreated plants: **A** Mean temperature. **B** Relative humidity. **C** Maximum temperature

Figure 3 shows significant associations in open field between *P. longifila* infestation and climatic parameters in both treated plants (A) and untreated plants (B), highlighting the influence of the environment on the population dynamics of the pest.

In the treated plants, infestation showed a moderate positive correlation with average temperature (R² = 0.43, *p* < 0.05), indicating that temperature ranges favored infestation. Relative humidity also showed a positive association, although of lesser magnitude (R² = 0.19, *p* < 0.05), showing that humidity favors infestation. However, for maximum temperature, extreme values ​​did not show a significant correlation (R² = 0.02, *p* > 0.05).

In untreated plants, infestation also showed a positive correlation with average temperature, although of a smaller magnitude compared to treated plants (R² = 0.12, *p* < 0.05). In contrast, relative humidity showed a weak but positive association (R² = 0.20, *p* < 0.05), indicating that this factor favors the pest in the absence of control measures. Furthermore, maximum temperature showed a weak but positive relationship (R² = 0.10, *p* < 0.05), suggesting that higher temperatures could favor infestation in untreated plants.

The comparison between open field and greenhouse showed highly significant contrasts in infestation for all climatic parameters evaluated, in both treated and untreated plants, although with opposite response patterns between the two groups (Table 3).

**Table 3:** Comparison of infestation in treated and untreated plants between environments estimated using GLMM

Contrast open field - greenhouse	Estimate	SE	Z Value	*p*-Value
Treated plants				
Mean temperature	0.97	0.16	5.94	0.0001 **
Relative humidity	0.98	0.15	6.50	0.0001 **
Maximum temperature	0.97	0.17	5.71	0.0001 **
Untreated plants				
Mean temperature	-3.16	0.42	-7.50	0.0001 **
Relative humidity	-2.35	0.30	-7.97	0.0001 **
Maximum temperature	-1.49	0.23	-6.39	0.0001 **

The values ​​are on a logarithmic scale. SE corresponds to the standard error

Significance levels: ** *p* < 0.001 (highly significant)

In open field, infestation in treated plants was 2.63 times higher than in greenhouse, both when average temperature and maximum temperature were considered, differences that were highly significant (*p* < 0.0001). Similarly, infestation associated with relative humidity was 2.67 times greater in open field than in greenhouse, a difference that was also highly significant (z = 6.50; *p* < 0.0001). (Table 3).

In open field, a 1 °C increase in average and maximum temperature was associated with an approximate 14% reduction in infestation. However, this effect was not statistically significant, as the 95% confidence interval included the value of no effect, indicating a weak negative trend with high variability caused by fluctuating infestations resulting from the effectiveness of sprays and continuous recolonization by the pest. In contrast, a 1% increase in relative humidity interacted with an approximate 0.7% increase in field infestation, although this effect was also not statistically significant, with confidence intervals that included the null value (Table 4).

**Table 4:** Estimated slopes of Climatic parameter effects by environment in treated plants

Greenhouse				Open field			
Parameter	Trend	SE	IC 95%	Parameter	Trend	SE	IC 95%
Mean temperature	-0.03	0.14	-0.31, 0.24	Mean temperature	-0.15	0.114	-0.38, 0.07
Relative humidity	0.06	0.05	-0.04, 0.17	Relative humidity	0.01	0.029	-0.05,0.07
Maximum temperature	0.05	0.06	-0.07, 0.16	Maximum temperature	-0.14	0.073	-0.29, 0.00

The values ​​are on a logarithmic scale. SE corresponds to the standard error

In the greenhouse, the thermal effect was even smaller, with an estimated 3% decrease in infestation for every 1 °C increase, accompanied by wide confidence intervals that included a value of no effect. This suggests that the average temperature did not significantly explain the variation in infestation under protected conditions. Regarding relative humidity, a more pronounced positive association was observed, with an estimated 7% increase in infestation for every 1% increase; however, this effect was also not statistically significant and presented a high degree of uncertainty. Finally, the comparison of the temperature and relative humidity slopes between the field and the greenhouse showed no significant differences for any of the parameters evaluated (*p* > 0.54) (Table 4).

The Binomial Hurdle Model analysis showed that, in untreated plants, the probability of infestation was consistently lower in open field than in greenhouse. In relative terms, infestation was approximately 77% lower in open field considering the maximum temperature, 96% lower considering the average temperature, and 90.5% lower with respect to relative humidity; these differences were highly significant (Table 3).

Regarding the thermal effect, in open field, a 1 °C increase in the maximum temperature was associated with a significant increase in the probability of infestation, raising it by approximately 134%. Conversely, in greenhouse, the same temperature increase was associated with an estimated 19% increase, an effect that was not statistically significant and had wide confidence intervals (Table 5).

**Table 5:** Estimated slopes of Climatic parameter effects by environment in untreated plants

Greenhouse				Open field			
Parameter	Trend	SE	IC 95%	Parameter	Trend	SE	IC 95%
Mean temperature	-2.01	0.31	-2.62, -1.40	Mean temperature	3.21	0.62	1.99, 4.43
Relative humidity	0.43	0.11	0.22, 0.64	Relative humidity	-0.46	0.11	-0.67, -0.25
Maximum temperature	0.17	0.11	-0.04, 0.38	Maximum temperature	0.85	0.27	0.32,1.38

The values ​​are on a logarithmic scale. SE corresponds to the standard error

In open field, a 1 °C increase is associated with a very marked increase in the probability of infestation, multiplying it by approximately 25, a highly significant effect. Conversely, under greenhouse conditions, a 1 °C increase in average temperature is associated with an approximately 87% reduction in the probability of infestation, also a statistically significant effect (Table 5).

Regarding relative humidity, in open field, a 1% increase was associated with a significant reduction of approximately 37% in the probability of infestation, while in greenhouse, the same increase resulted in an increase of nearly 54%, also statistically significant. The comparison of slopes confirmed that there were no differences in maximum temperature, but there were differences in mean temperature and relative humidity between the environments (*p* < 0.0001), indicating that the infestation response was not only greater in magnitude, but also of opposite sign between open field and greenhouse (Table 5).

### Effectiveness of phytosanitary applications

To control *P. longifila* throughout the growing cycle, 7 treatments were carried out in a greenhouse and 16 in open field. The efficacy of the evaluated pesticides varied between the two cultivation systems (Table 6).

**Table 6:** Estimation of pesticide efficacy using GLMM (Tweedie) on infestation in the two cropping systems

Pesticides Used in the Open Field	Efficacy (%)	Pesticides Used in the Greenhouse	Efficacy (%)
*Azadirachta indica*	49% a	*Azadirachta indica*	51% b
Abamectin	62% a	Abamectin	78% ab
Spinetoram	72% a	Spinetoram	72% ab
Imidacloprid	84% a	Thiamethoxam + lambdacyhalothrin	100% a
Thiamethoxam + lambdacyhalothrin	51% a		
Acetamiprid	82% a		

Different lowercase letters indicate statistically significant differences among treatments (Tukey’s HSD test, *p* < 0.05)

In the greenhouse, efficacy ranged from 51% to 100%. The treatment with thiamethoxam + lambda-cyhalothrin achieved the highest efficacy at 100%, followed by abamectin and spinetoram at 78% and 72%, respectively. *A. indica* showed the lowest efficacy at 51% against *P. longifila* infestation.

In open field, efficacy values ​​ranged from 49% to 84%, with no statistically significant differences between products (*p* > 0.05). However, the highest levels of control were observed with imidacloprid (84%) and acetamiprid (82%), followed by spinetoram (72%) and abamectin (62%). Treatments with *A. indica* and the thiamethoxam + lambda-cyhalothrin mixture showed lower efficacy, although statistically comparable to the others.

Analysis using Tweedie’s GLMM model revealed a significant effect of temperature on pesticide efficacy (Table 7). In the case of abamectin, both the average and maximum temperatures showed a negative correlation with its efficacy, indicating that each 1 °C increase reduces its effectiveness in controlling *P. longifila* by approximately 42%.

**Table 7:** Effect of average and maximum temperature on pesticide efficacy

Pesticides	Climatic Parameters	Coefficient (β)	Exp (β)	Percentage change in efficiency per 1 °C increase	*p*-value
Abamectin	Meam temperature	0.546	1.725	− 42%	0.0128 *
*A. indica*	Meam temperature	-0.732	0.481	+ 108%	0.0110 *
Abamectin	Maximum temperature	0.540	1.714	− 42%	0.0053 *
*A. indica*	Maximum temperature	-0.659	0.517	+ 94%	0.0267 *

Coefficients (β) estimated using Tweedie GLMM with log link. Exp(β) indicates the proportional change in efficacy per additional degree of temperature

Significance levels: * *p* < 0.05 (significant)

Conversely, *A. indica* showed a significant increase with temperature. Each 1 °C increase in average or maximum temperature improved its efficacy by 100% and 94%, respectively. The temperature-induced increase in the efficacy of *A. indica* could explain the phytotoxicity and burns observed in the crop under greenhouse conditions. Due to these adverse effects, *A. indica* was excluded from the Integrated Pest Management (IPM) program.

### Population dynamics and monitoring

#### Infestations percentages in treated and untreated plants

**Fig. 4:**
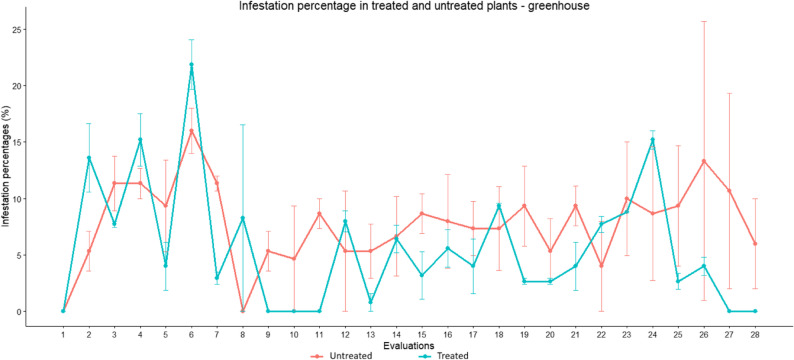
Temporal variation of infestation percentages in treated and untreated plants under greenhouse conditions

Figure 4 shows the temporal variation of infestation percentages at different evaluation dates under greenhouse conditions. Infestation levels fluctuated over time, and overlapping error bars were observed between treated and untreated plants at several evaluation dates. Despite this temporal variability, untreated plants maintained, and continue to maintain, higher populations of *P. longifila* over time, while treated plants showed significant reductions in infestation percentages due to pesticide applications made after exceeding the treatment threshold. In untreated plants, the tolerable infestation threshold was exceeded in six evaluations, with values ​​ranging from 10.7% to 16%.

**Fig. 5:**
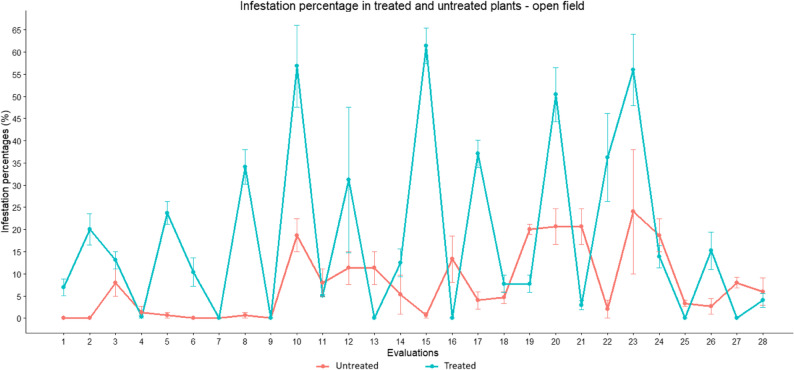
Temporal variation of infestation percentages in treated and untreated plants under open field conditions

Figure 5 illustrates the variation in infestation percentages among the open-field assessments. Infestation levels showed marked temporal fluctuations, with overlapping values ​​between the treated and untreated plants groups at various times. In general, untreated plants tended to maintain lower infestation percentages over time. In untreated plants, infestation levels ranged from 11.3% to 20.7%. However, severe damage to the shoot tips and buds was observed in untreated plants, limiting vegetative growth and preventing the maintenance of stable pest populations over time.

#### Larvae determination in greenhouse and open field

The number of larvae per plant is significantly higher in open field than in greenhouse. The average number of larvae counted in the greenhouse was 7, while the average number of larvae counted per plant in open field was 19.

**Fig. 6:**
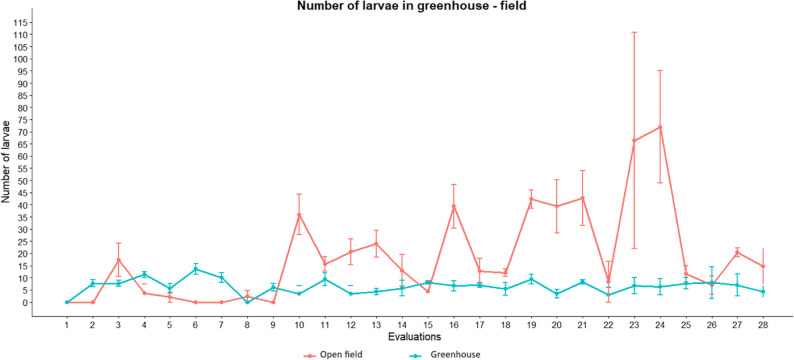
Number of larvae on untreated plants in the greenhouse and open field

This reflects that the greenhouse had the lowest averages, while the field had higher averages. Furthermore, the unviability of the shoots due to the severity of the damage made it difficult to sustain the larvae (Fig. 6).

**Fig. 7:**
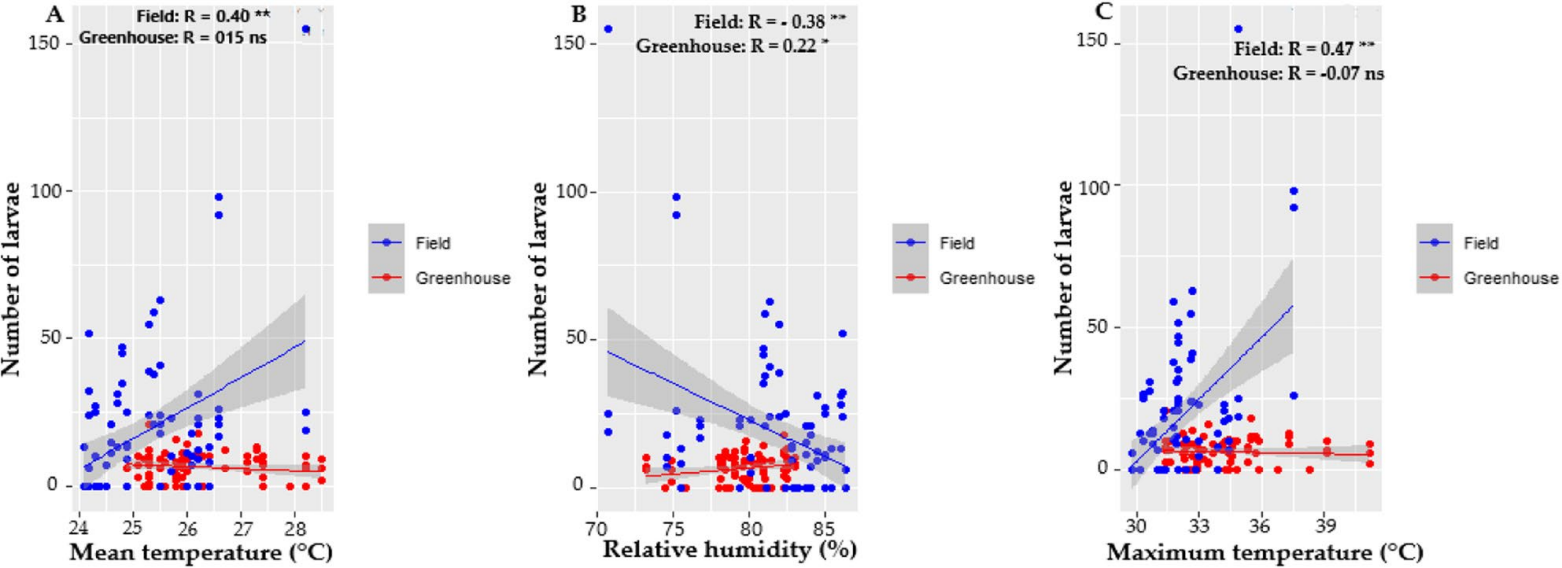
Relationship between climatic factors and the number of larvae on untreated plants in the greenhouse and open field, mean temperature (**A**), relative humidity (**B**) and maximum temperature (**C**).

Figure 7 shows the correlations between the number of larvae and climatic parameters in both environments. In open field, the three variables studied showed statistical significance, where average and maximum temperatures reflected a positive influence on the number of larvae counted, while relative humidity showed a negative influence on the larvae. In contrast, the relative humidity in the greenhouse favored *P. longifila*.

**Fig. 8:**
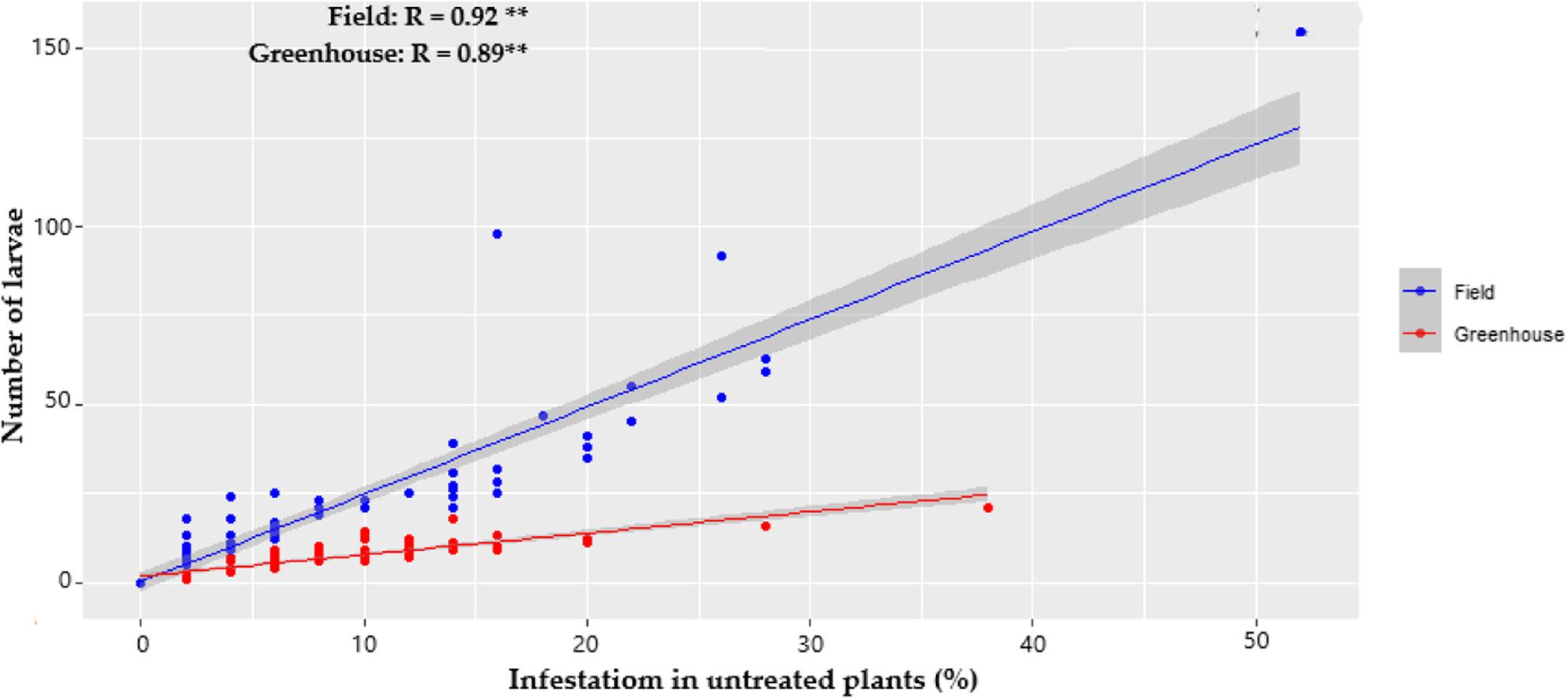
Relationship between infestation percentage on untreated plants and the number of larvae per shoot in the greenhouse and open field

Figure 8 shows a positive and highly significant correlation between infestation and the number of larvae counted in both environments (*R* = 0.92 in the open field and *R* = 0.89 in the greenhouse). However, the slopes differed markedly: in the open field it was 2.46, while in the greenhouse it was 0.6. This indicates that, with an increase in the number of larvae, the percentage of infestation increases more rapidly in the open field than in the greenhouse. Biologically, this difference is because environmental conditions in the open field favor more aggressive larval development or greater damage, while the progression of infestation is slower in the greenhouse. Consequently, the populations of *P. longifila* in the greenhouse remained below critical damage levels.

Analysis using GLMM models with a Tweedie distribution, with coefficients transformed from the logarithmic scale to the original response scale, consistently showed that the average number of larvae was significantly higher in the open field than in the greenhouse. Specifically, the open field system showed values ​​approximately 3.16 times higher than those of the greenhouse considering the average temperature, 3.5 times higher under the effect of the maximum temperature, and 2.18 times higher in relation to relative humidity (Table 8).

**Table 8:** Comparison of the number of larvae between environments estimated by GLMM

Contrast open field - greenhouse	Estimate	SE	Z Value	*p*-Value
Mean temperature	1.15	0.17	6.879	0.0001 **
Relative humidity	0.78	0.22	3.482	0.0005 **
Maximum temperature	1.25	0.17	7.307	0.0001 **

The values ​​are on a logarithmic scale. SE corresponds to the standard error

Significance levels: ** *p* < 0.001 (highly significant)

In open field, a 1 °C increase in average temperature was associated with an approximately 53% increase in the number of larvae, a statistically significant effect. Similarly, a 1 °C increase in maximum temperature was associated with an approximately 32% increase in the number of larvae, also a significant effect. In contrast, in greenhouse, temperature increases showed weaker effects, with estimated increases of 10% for average temperature and 5% for maximum temperature, neither of which was statistically significant (Table 9).

**Table 9:** Slopes of the effect of Climatic parameters on larval number

Greenhouse				Open field			
Parameter	Trend	SE	IC 95%	Parameter	Trend	SE	IC 95%
Mean temperature	0.09	0.16	-0.21, 0.40	Mean temperature	0.43	0.12	0.20, 0.66
Relative humidity	-0.16	0.11	-0.38	Relative humidity	-0.07	0.04	-0.14, -0.00
Maximum temperature	0.05	0.07	-0.08, 0.18	Maximum temperature	0.28	0.07	0.15, 0.41

The values ​​are on a logarithmic scale. SE corresponds to the standard error

Regarding relative humidity, in open field, a 1% increase was associated with an approximate 7% decrease in the number of larvae, a statistically significant effect. In greenhouse, a 1% increase in relative humidity was associated with an estimated 15% reduction in the number of larvae, although this effect was not statistically significant (Table 9).

Comparison of the slopes between environments did not indicate significant differences in the response of the number of larvae to average temperature (*p* > 0.10) or relative humidity (*p* > 0.49). However, a significant difference was detected between open field and greenhouse for maximum temperature (*p* < 0.0186), demonstrating that the influence of this thermal parameter on the number of larvae was considerably greater in open field than in the protected system.

#### Estimation of populations through monitoring with chromatic traps

**Fig. 9:**
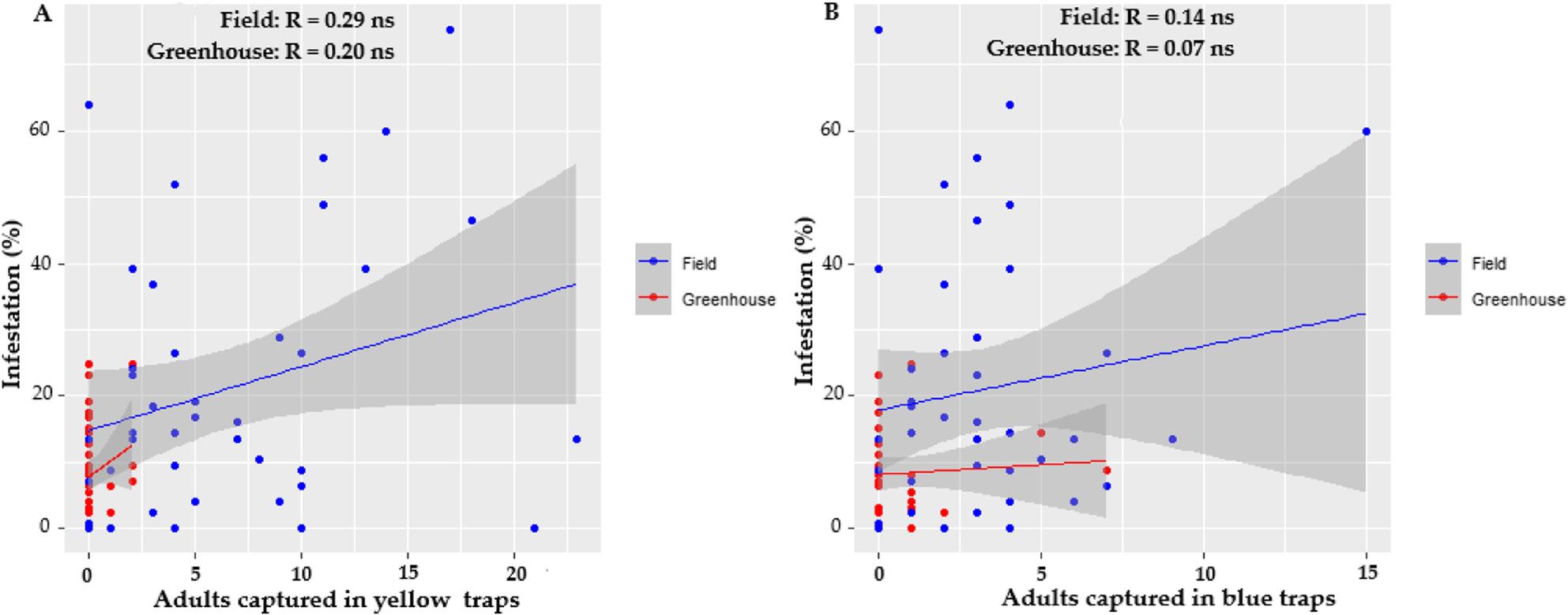
Correlation between infestation percentage and the number of adults captured in traps in open fields and greenhouses: (**A**) captures in yellow traps; (**B**) captures in blue traps

Chromatic traps can serve as a rapid and effective tool to quantify pest infestations and support management decisions. To assess their reliability for monitoring *P. longifila* populations, the correlation between infestation percentages and the number of adults captured in yellow and blue traps was evaluated in both environments (Fig. 9). The correlation between infestation percentage and the number of adults captured in yellow traps was positive in both the open field (*R* = 0.29) and greenhouse (*R* = 0.20), but not statistically significant (*p* > 0.05). Similarly, correlations with blue traps were weak and non-significant in both the open field (*R* = 0.14) and greenhouse (*R* = 0.07). Considering that treatment decisions in this study were based on an action threshold of 10% infestation, an additional correlation analysis was conducted to assess trap performance at different infestation levels. When infestation levels exceeded 10%, the correlation with yellow traps increased slightly in the open field (*R* = 0.35) and greenhouse (*R* = 0.18), compared with blue traps (*R* = 0.14 and *R* = 0.10, respectively). However, when infestation levels were below 10%, the correlations weakened further—yellow traps in open field (*R* = − 0.08) and greenhouse (*R* = 0.25); blue traps in open field (*R* = 0.09) and greenhouse (*R* = 0.13). None of these relationships were statistically significant (*p* > 0.05).

These findings suggest that while, color traps detect the presence of *P. longifila*, they are not a good tool for estimating the abundance of *P. longifila*, which limits their usefulness as a monitoring tool.

## Discussion

The climate signal regarding infestation in treated plants was weak and highly variable, both in open field and in greenhouse.

The application of statistical models based on GLMM and GAM allowed for the control of climatic covariates and the capture of potential nonlinear relationships, demonstrating that the cropping system exerted a consistent and highly significant effect on the baseline infestation level. In particular, infestation in treated plants was between 2.63 and 2.67 times higher in open field than in greenhouse, regardless of the climatic parameter considered. This indicates that the temporal variation of infestation was strongly modulated by structural and management factors, such as the transient effectiveness of sprays and the continuous recolonization of the pest, which can mask or weaken the climatic signal. In this context, the absence of significant differences in slopes between open field and greenhouse reinforces the idea that climate did not substantially modify the sensitivity of the infestation, but rather acted on different baseline levels determined primarily by the cropping system.

In untreated plants, the cultivation system decisively modulated both the baseline probability of infestation and the way climatic variables influenced it. The consistently lower probability of infestation observed in open field, with reductions of up to 96% compared to greenhouse, does not necessarily reflect lower biological pressure from the insect, but was strongly conditioned by the availability of susceptible plant tissue. In open field, untreated plants experienced a rapid loss of tender shoots as a consequence of accumulated damage, generating prolonged periods without a suitable food source for the larvae. This structural limitation not only restricted the establishment of *P. longifila*, but also reduces the suitability of the host plant for other herbivorous insects [34]. This structural limitation of the host explains the high frequency of zeros observed in the data and justifies the use of the Hurdle-Binomial model, which allows explicit discrimination between the occurrence of infestation and its intensity when the resource is available.

Under greenhouse conditions, untreated plants maintained more continuous shoot production, which favored the persistence of the infestation once established. Furthermore, the physical protection of the system restricted dispersal and recolonization processes from the outside, reducing the variability associated with population entry and exit. As a result, the greenhouse infestation exhibited a significantly higher baseline probability and a more stable climatic response, reflecting a system in which host availability and pest persistence were not limited by resource depletion.

This structural contrast between systems allows for the interpretation of the observed thermal responses. In open field, the increase in maximum and average temperatures was associated with a marked increase in the probability of infestation when plant tissue was available, suggesting that temperature acted as an amplifying factor for recolonization and the temporary establishment of the pest during brief periods of host availability. In particular, the extremely intense effect of average temperature in open field could reflect a synchronization between favorable thermal conditions, localized plant regrowth, and the arrival of new individuals from surrounding areas.

Conversely, in greenhouse, the negative infestation response to increased average temperature suggests that, once host availability is assured, elevated temperatures could exceed the optimal ranges for insect development or survival, or interact with plant physiological factors that reduce tissue suitability for oviposition or larval development. This opposing response, clearly evidenced by the highly significant differences in slopes between the environments, demonstrates that the protected microclimate not only modifies the magnitude of the infestation but also the direction of the climatic response.

Taken together, these results indicate that, in untreated plants, infestation dynamics were determined by a complex interaction between climate, host availability, and system connectivity. While in open fields infestation was intermittent and strongly influenced by shoot loss and continuous recolonization, in greenhouse physical protection and host stability fostered a scenario of greater population persistence. This contrast highlights the importance of specifically considering the structure of the cropping system and the generation of structural zeros when analyzing pest responses to climatic variables, particularly in studies aimed at understanding risks under climate variability and change scenarios.

These patterns are consistent with previous research. For instance, Castillo et al. [35], indicates that temperatures above 30 °C or below 11 °C, especially when combined with daily fluctuations greater than 10 °C, limit the development of *P. longifila*. Similarly, European Food Safety Authority et al. [36] indicates that this species thrives in warm, humid climates (60–98% relative humidity) and is negatively affected by temperatures outside the optimum range of 11–28 °C. In this context, our results provide empirical support for these thermal thresholds under contrasting production systems. Although greenhouse temperatures reached higher maximum values (34.4 °C vs. 32.1 °C in the field) and slightly higher minimum temperatures (21.6 °C vs. 20.2 °C), physical exclusion through netting effectively restricted adult entry and recolonization, thereby reducing infestation levels. In contrast, in open-field conditions, direct exposure to environmental variability and continuous recolonization from surrounding areas favored pest persistence.

The observed responses to relative humidity further support this system-dependent interpretation. Consistent with our results, Hernandez et al. [11] reported that *P. longifila* tolerates a wide thermal range (22–38.7 °C), whereas its response to humidity is more variable. Mena et al. [37] observed a negative correlation between infestation levels and high relative humidity (79.9–81.8%), whileValarezo et al. [7] showed that increased humidity reduces infestation and larval viability, likely due to osmotic stress affecting larval water balance and physiological function [38]. Relative humidity reinforced this pattern of structural dependence within the system. In open field, increased humidity was associated with a lower probability of infestation, possibly related to higher larval mortality, interference with oviposition, or indirect effects stemming from less favorable conditions for recolonization. Conversely, in greenhouse, the combination of high humidity and environmental stability significantly favors infestation, consistent with an environment that promotes the pest’s survival and continued development.

Taken together, both our findings and previous evidence suggest that microclimate management represents a viable preventive strategy within Integrated Pest Management (IPM) programs. The presence of potentially genetically distinct lineages [39], combined with the high reproductive capacity of *P. longifila* (21–33 generations per year) [40], may contribute to population stability in greenhouse systems. Under this scenario, regulating microclimatic conditions—maintaining moderate temperatures (24–30 °C), avoiding daily fluctuations exceeding 10 °C, reducing relative humidity to approximately 75–80%, and optimizing ventilation—emerges as a practical approach to limiting severe outbreaks.

The implementation of Integrated Pest Management (IPM) strategies in tomato cultivation is essential for its sustainable production, particularly in regions where *P. longifila* poses a significant threat. In this context, it is crucial to explore alternatives such as protected cultivation, pest monitoring, and integrated management. Van Lenteren [41] emphasizes that IPM is a reliable and economically viable strategy, widely adopted in greenhouse crops globally. Furthermore, it is possible to have lower pest pressure and a greater number of natural enemies if they are managed with the least amount of chemicals possible [42].

While this study analyzes the short-term effects of pesticides on *P. longifila* infestation, it is essential to consider the long-term implications. Excessive pesticide use can induce pest resistance and negatively affect natural enemies, compromising the effectiveness of chemical control. Zhang et al. [43] point out that this practice is common among farmers due to a lack of knowledge in pest and disease management.

In greenhouse, pest management requires a more rigorous approach due to the potential accumulation of pesticide residues and the greater sensitivity of the enclosed environment. Previous studies have indicated that residues tend to concentrate at higher levels in greenhouses [44], and that improper pesticide application can compromise product quality and increase the presence of chemical residues [45]. Furthermore, ineffective pesticide management affects food safety, making the optimization of pesticide use in both greenhouses and open fields crucial [46]. Therefore, following the principles of Integrated Pest Management (IPM), the use of low-toxicity insecticides and targeted applications based on infestation levels were prioritized.

Our results show that greenhouse-treated plants consistently exhibited lower infestation levels than untreated plants, confirming the effectiveness of chemical applications in this controlled environment. Specifically, abamectin demonstrated superior efficacy, ranging from 71% to 100% with initial infestations of 10% to 15%, while spinetoram achieved 87% efficacy with infestations near 22%, and the combination of thiamethoxan + lambda-cyhalothrin achieved 90% efficacy when infestation levels reached 10% and was applied as part of a chemical group rotation strategy. These data suggest that greenhouse applications should be prioritized when infestation levels approach 10–15%, the range in which insecticide efficacy is at its maximum and the expansion of the *P. longifila* population is prevented.

The use of *A. indica* as a botanical insecticide, while effective at some infestation levels (44–100%) and enhanced by temperature, caused phytotoxicity and leaf burn in plants, leading to its exclusion from the IPM program. Authors such asNiwas and Palmar [47] highlight the biocidal efficacy of this product but warn that high concentrations can cause phytotoxicity and growth retardation. This effect is due to its high concentration of azadirachtin (4 g/L) and compounds such as diacetylated limonoids, salannins, melantriol, and nimbedinins, which induce paralyzing, antifeedant, and repellent effects on the pest [25]. In the present study, it was also observed that the effectiveness of spinetoram decreased when applied after the use of *A. indica*, probably due to its antifeedant effect, which reduces the exposure of *P. longifila* to the insecticide.

Regarding the influence of temperature on *A. indica*, efficacy increased by 94–100% for each 1 °C increase in temperature, likely due to rapid solvent evaporation and product concentration on the leaf surface. Previous studies have shown that 50% concentrations of *A. indica* cause leaf necrosis and growth inhibition in tomatoes, while concentrations of 5–10% do not show significant negative effects [48]. However, Niwas and Palmar [47] reported that concentrations of 2–3% cause seedling burns and growth delays in squash. Although the formulation used in this study was within the safe range (4%), information on the phytotoxicity of *A. indica* is varied. Therefore, it is important to consider temperature when establishing more precise strategies within the greenhouse, given the high radiation and temperature in protected systems where ventilation is limited.

Spinetoram, a concentrated suspension insecticide from the spinosyn class [49], showed high efficacy in controlling *P. longifila*, consistent with previous studies on its effectiveness in managing *Liriomyza* spp [50]. This highlights its potential as a key component in IPM programs, especially for moderate infestations, allowing for a reduction in reliance on broad-spectrum insecticides and minimizing environmental impact.

While the temperatures recorded inside the greenhouse (34.4–41.1 °C) negatively affected the effectiveness of abamectin, the efficacy of the applications remained within an acceptable range under low infestation conditions. Abamectin is a compound highly sensitive to ultraviolet radiation and high temperatures, factors that accelerate its photochemical degradation and reduce its persistence in foliage [51]. Furthermore, it has been documented that *Liriomyza trifolii* can develop adaptive cross tolerance to abamectin under heat stress, suggesting that high temperatures could enhance physiological detoxification mechanisms and decrease the insect’s susceptibility to treatment [52].

Despite these limitations, abamectin exhibits low toxicity to beneficial arthropods in open fields due to its short environmental stability, rapid absorption by treated plants, and the fast degradation of surface residues [53]. Taken together, these findings explain the observed reduction in product efficacy under greenhouse conditions with extreme temperatures. Nevertheless, abamectin remains a favorable alternative within Integrated Pest Management (IPM) programs, thanks to its relatively low impact on beneficial insects. However, its application should be carried out with caution and under close monitoring, considering the potential differentiated effects on beneficial organisms and the influence of thermal conditions on their persistence and efficacy.

In open field, the persistence of *P. longifila* and high infestation levels required weekly pesticide applications, totaling 16 treatments with various plant protection products. Efficacy varied widely depending on the initial infestation level, highlighting the importance of adjusting applications according to the treatment threshold. For *A. indica*, efficacy was inconsistent, ranging from 0% to 100% against initial infestations of 7–15%, suggesting limited stability under high pest pressure. Abamectin, on the other hand, showed efficacy of 35% and 100% for initial infestations of 20% and 10%, respectively, indicating that its optimal performance is achieved when applications are made at early stages of population development. Similarly, spinetoram achieved efficacies of up to 100% at moderate infestations (24–34%), while acetamiprid and imidacloprid maintained high effectiveness (> 90%) even against heavy infestations (50–60%), demonstrating their potential to suppress severe outbreaks of *P. longifila*. In contrast, thiamethoxam + lambda-cyhalothrin showed a variable response (0–100%), possibly associated with cross-resistance or loss of efficacy under high population pressure, as increases in infestation were observed after its application. Therefore, its use should be managed with caution within active ingredient rotation programs. Accordingly, Kroschel et al. [54] point out that frequent insecticide application can favor pest proliferation by disrupting the natural balance of the agroecosystem and reducing the presence of natural enemies.

Authors such as Constante et al. [55] and Chirinos et al. [56] documented the indiscriminate use of pesticides in the management of this pest and agree that its control is predominantly chemical, with the frequent use of highly toxic insecticides such as methamidophos and methomyl. Cardona et al. [57] recommend rotating products such as thiamethoxam + lambda-cyhalothrin, imidacloprid, and abamectin, with reported efficacies of 62.92%, 59.67%, and 34.27%, respectively. However, Goldsmith et al. [40] and Kroschel et al. [54] suggest that selective treatments with low doses of imidacloprid, as well as the use of non-selective insecticides such as sulfur, rotenone, and chlorpyrifos, can effectively reduce *P. longifila* when applied correctly. These findings are consistent with our results, as the recommended doses of imidacloprid reduced infestation by 91% in extreme infestations.

The low efficacy of *A. indica* in open field could be due to its poor chemical stability, rapid environmental degradation, and short pest life cycles that allow for rapid colonization. Previous research indicates that formulations with high concentrations of azadirachtin can reduce the presence of synergistic compounds that enhance their insecticidal effect [58]. Kroschel et al. [59] reported that *A. indica* extracts have a repellent effect but with a biological persistence of three days. Other studies confirm that the half-life of azadirachtin ranges from 1.84 to 4.53 days, with degradation of the active ingredient between 96% and 98% when the temperature reaches 54 °C [58]. Furthermore, Kumar and Parmar [60] emphasize the stability of commercial formulations containing *A. indica* stored at room temperature (12 –33 °C) because it degrades between 59.52% and 81.27% of the active ingredient, resulting in formulation losses. This could explain the limited efficacy of the product used in this study.

The low level of infestation observed in the greenhouse is primarily attributed to the use of anti-aphid mesh, which acted as an effective physical barrier preventing the entry of adult *P. longifila*. The mesh used had a density of 26 × 40 filaments per square inch (0.98 × 0.64 mm), a size smaller than that of adult *P. longifila*, which measures approximately 1.03 mm, as reported by Valarezo et al. [7] Although these authors observed infestation levels between 8% and 18% in tomato crops covered with shade mesh, indicating that physical exclusion does not eliminate the risk of infestation, the results of this study confirm that aphid netting is an effective tool within Valarezo et al. [7] integrated pest management. Its implementation in greenhouses could significantly reduce pest pressure without relying exclusively on insecticides, promoting a more sustainable and environmentally responsible production system.

Larval counts reinforce the idea that the cultivation system not only influences the probability of infestation but also the magnitude of the population response once the pest is established. Larval counts were consistently higher in open field, with values ​​two to more than three times higher than those recorded in greenhouse. This indicates that, when plant tissue is available, outdoor conditions favor greater larval accumulation. This pattern suggests that open systems allow for greater spatial connectivity and continuous recolonization from surrounding areas, increasing the flow of individuals and amplifying population pressure on host plants.

In open field, the number of larvae responded positively to increases in mean and maximum temperatures, indicating that these parameters acted as factors promoting larval development and population dynamics. The significant increase in larval abundance associated with the temperature increase suggests that higher temperatures favor key processes such as growth, survival, and possibly oviposition rate, resulting in quantifiable increase in population size. Conversely, under greenhouse conditions, the thermal effects were weak and not significant, suggesting that the microclimatic stability of the protected system dampens the direct influence of temperature variations on larval abundance. The differential effect of maximum temperature between environments is particularly relevant. The greater sensitivity of larval numbers to this parameter in open fields indicates that temperature spikes, frequent in outdoor systems, could play a disproportionate role in population amplification. Conversely, in greenhouse, the attenuation of extreme temperatures and partial control of the microclimate appear to limit the ability of maximum temperature to induce similar population responses.

Relative humidity showed a negative effect on larval numbers in both systems, although this effect was only statistically significant in the open field. This pattern suggests that increased humidity could negatively affect larval survival or the quality of the immediate microenvironment, especially in open conditions, where the direct interaction between food availability and the microclimate could intensify this effect. In the greenhouse, the absence of a significant effect of relative humidity could be associated with greater environmental stability and less exposure to external mortality factors.

Regarding the influence of climatic parameters on larval numbers, our results indicate that temperature had a positive effect under open-field conditions, whereas high relative humidity exerted a negative influence on larval development. In contrast, under greenhouse conditions, relative humidity tended to favor larval survival. These findings partially coincide with those reported by Valarezo et al. [7], who observed a negative correlation between relative humidity and the number of viable larvae. Additionally, Duque et al. [12] pointed out that high larval densities in tomato crops can facilitate the persistence and spread of *P. longifila*, increasing infestation levels even in industrial areas. In this context, Valarezo et al. [7] emphasized the importance of quantifying larval numbers as a more accurate estimate of the actual insect population. Taken together, these results indicate that, once the establishment barrier was overcome, the dynamics of larval numbers were strongly influenced by the interaction between climate and the cultivation system. While in open fields, temperature variations, particularly maximum temperatures, acted as key drivers of larval abundance, in greenhouses, microclimatic stability tended to decouple the population response from environmental fluctuations. This contrast underscores the importance of considering not only the occurrence of infestation but also population intensity and its differential sensitivity to climate when designing integrated management strategies in open and protected systems.

Our results indicate that monitoring with yellow and blue chromatic traps is not a reliable method for estimating the abundance of *P. longifila*, as no significant correlation was found between adult capture and the level of infestation in the plants, even under high infestation conditions. However, previous studies have shown variations in the effectiveness of this type of trap. Musto and Martos [61] reported that yellow panels with white light were the most efficient treatment for capturing adults in tomato, while Shi et al. [62] mention that light traps increase captures. Despite this, to date there are no specific studies analyzing the relationship between the percentage of infestation and the capture of adults in chromatic traps for *P. longifila*. In contrast, for other pests such as *Bemisia tabaci* in cotton, Karut and Kazak [63] did demonstrate a significant correlation between captures in chromatic traps and infestation levels in the field. Therefore, while chromatic traps can be useful as a complementary monitoring tool, their effectiveness in predicting *P. longifila* infestations has not yet been demonstrated, and their use should be integrated with direct sampling strategies and field observations.

This study demonstrates that environmental conditions significantly influence the population dynamics of *P. longifila*, with clear differences observed between greenhouse and open-field cultivation. While pesticide use resulted in a temporary reduction in infestation, the lack of a significant correlation between captures in sticky traps and plant damage levels indicates that this monitoring method alone is insufficient and should be complemented with direct sampling strategies. Considering the risk of resistance associated with repeated insecticide use, it is essential to strengthen Integrated Pest Management (IPM) programs by incorporating cultural practices, constant monitoring, and appropriate rotation of modes of action. Furthermore, future research should evaluate new active ingredients and selective combinations to optimize treatment efficacy and reduce dependence on chemicals, promoting more sustainable and resilient management of *P. longifila*.

## Conclusions

Infestation by *Prodiplosis longifila* differed significantly between greenhouse and open field systems. In open fields, infestation levels reached high values in both treated and untreated plants; however, populations could not be sustained over time due to the severe damage caused by larval feeding and the consequent loss of susceptible plant tissue. Pesticide efficacy was strongly dependent on infestation intensity: *A. indica* was effective at low infestation levels (10–15%), while abamectin and spinetoram showed high efficacy under moderate infestations (20–34%). At infestation levels between 31 and 37%, the combination of thiamethoxam + lambda-cyhalothrin was effective, although its use should be restricted to a maximum of two consecutive applications to avoid loss of efficacy. Under extreme infestation levels (50–61%), acetamiprid and imidacloprid provided the highest levels of control.

In greenhouse conditions, infestation levels were significantly lower and more stable, mainly due to physical exclusion provided by aphid-proof netting and the buffering effect of a controlled microclimate. Under these conditions, the biorational insecticides spinetoram and abamectin showed high efficacy at infestation levels of 10–20%, supporting their use as viable alternatives within Integrated Pest Management (IPM) programs. However, applications of *A. indica* should be approached with caution, as symptoms of phytotoxicity were observed under high greenhouse temperatures.

Direct monitoring of larvae through visual inspection of shoots and apical meristems proved to be a more reliable indicator of actual infestation levels than chromatic traps, allowing for a more precise timing of control interventions. However, the population dynamics of *P. longifila* differed markedly among the cultivation systems, highlighting the need to adapt monitoring and management strategies to the specific structural and environmental conditions of each production system.

This study demonstrates that the use of greenhouses with bamboo structures and aphid-proof netting represents a sustainable and immediately applicable alternative for revitalizing tomato production on the Ecuadorian coast, providing effective mechanical protection and facilitating more efficient management of *Prodiplosis longifila* under real commercial conditions. These findings acquire additional relevance in light of the inclusion of *P. longifila* in the list of priority (quarantine) pests by the European Food Safety Authority (EFSA), highlighting the importance of preventive and containment-oriented strategies that reduce the risk of establishment and spread. By generating evidence under active management conditions, this study contributes practical insights that can be directly incorporated into Integrated Pest Management (IPM) programs, particularly those emphasizing physical exclusion, microclimate regulation, and rational insecticide use. Future research should evaluate the population dynamics of this pest over multiple production cycles and under different insecticide rotation schemes, further strengthening IPM strategies and supporting the development of more sustainable and resilient tomato production systems.

## Data Availability

Data is provided within the manuscript and there is no supplementary file.
